# The taxonomy of brain cancer stem cells: what's in a name?

**DOI:** 10.18632/oncoscience.25

**Published:** 2014-03-31

**Authors:** David H. Gutmann

**Affiliations:** ^1^ Department of Neurology, Washington University School of Medicine, St. Louis MO

**Keywords:** progenitor cell, glioma, ependymoma, astrocytoma, cell-of-origin, subventricular zone

## Abstract

With the increasing recognition that stem cells play vital roles in the formation, maintenance, and potential targeted treatment of brain tumors, there has been an exponential increase in basic laboratory and translational research on these cell types. However, there are several different classes of stem cells germane to brain cancer, each with distinct capabilities and functions. In this perspective, we discuss the types of stem cells relevant to brain tumor pathogenesis, and suggest a nomenclature for future preclinical and clinical investigation.

## INTRODUCTION

Brain cancers are the leading cause of cancer-related death in children and the fourth leading cause in adults [1-3]. Among the diverse histologic varieties of brain tumors, gliomas (glial cell neoplasms) comprise the most common subtype. Gliomas (or astrocytomas) are classified according to an established set of pathological features that define the four different malignancy grades, including low-grade (grades I and II) and high-grade (grades III and IV) tumors [4]. Unfortunately, there are limited effective therapeutic options available for these cancers, and individuals with these brain tumors experience significant morbidity and mortality [5-6]. While traditional anti-cancer therapies aim to kill rapidly dividing cells, complementary treatment strategies involve blocking the function of other cell types present in brain tumors [7]. As such, non-neoplastic astrocytes [8], blood vessels [9], and immune system-like cells (microglia and macrophages; [10-11]) each have been shown to participate in an instructive manner in glioma formation and progression. Leveraging this innate cellular heterogeneity, numerous studies have sought to define the individual contributions of these various stromal cell types to tumor formation and growth, leading to new brain tumor therapies [12-16].

**Figure 1:**
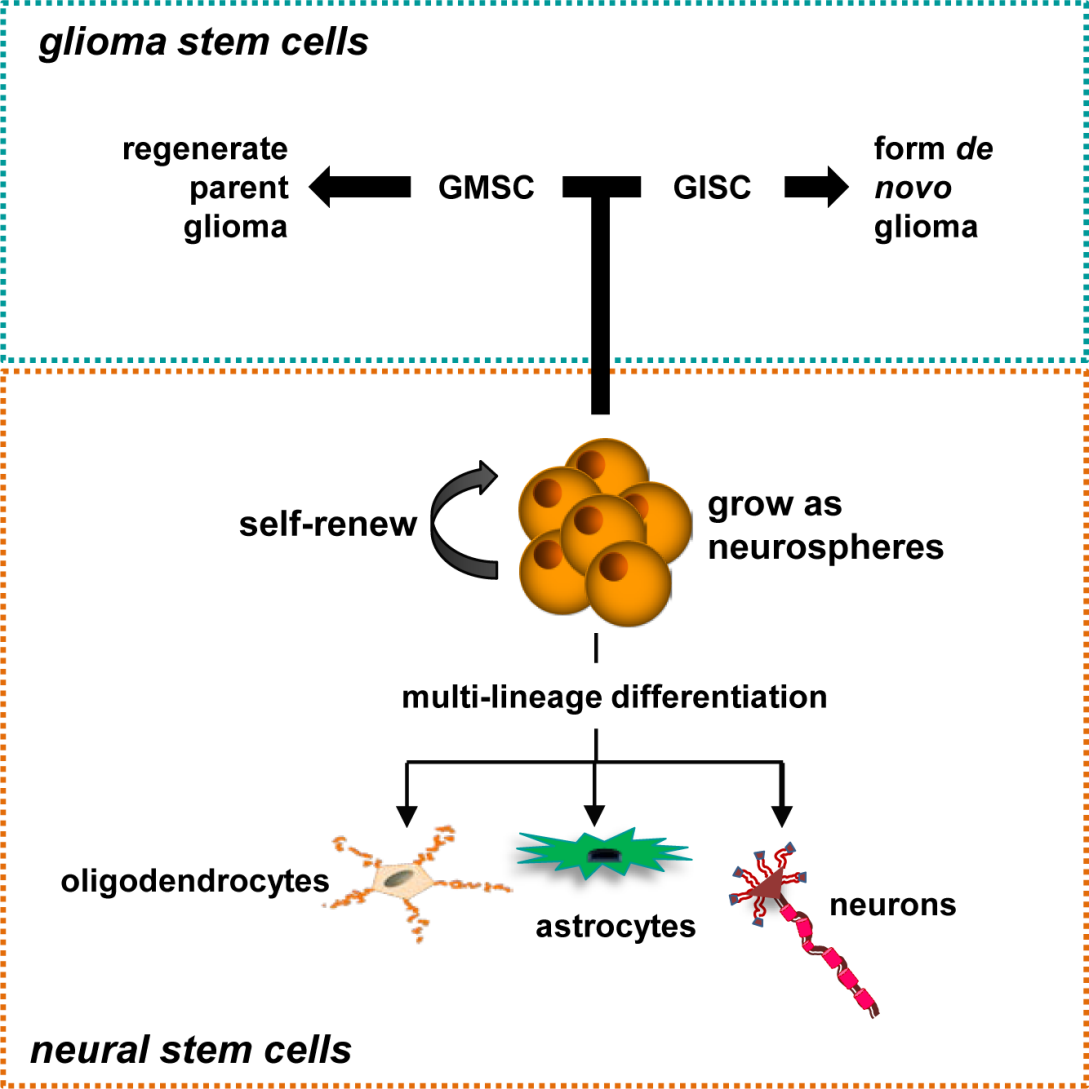
Stem Cells in Glioma Properties of neural stem cells include self-renewal, multi-lineage differentiation, and the ability to grow as neurospheres. GMSCs have the capacity to self-renew at limiting dilutions, undergo multi-lineage differentiation, grow as neurospheres, and in some instances, give rise to gliomas following transplantation into immunocompromised hosts. Similarly, GISCs are capable of forming glioma *in vivo* following the acquisition of glioma-causing genetic mutations.

Similarly, over the past decade, there has been an explosion in the number of publications describing the role of another of these cell types (stem cells) in the pathogenesis of glioma [17-18]. These investigations have revealed important functions for stem cells in glioma development, maintenance, and tumor heterogeneity. Because of their involvement in brain cancer, these stem cells are often collectively referred to as “cancer stem cells”, despite the fact that they may have different tissue origins, functions, and contributions to glioma biology. To further complicate matters, the criteria used to define these cancer stem cells in glioma often differ from study to study, and variably include the ability to self-renew (generate new stem cells), the capacity to form neurospheres, the expression of “stem cell” markers, the ability to give rise to all three major central nervous system (CNS) cell types (oligodendrocytes, astrocytes, and neurons), the capacity to proliferate for prolonged periods of time in culture, and the ability to generate gliomas following implantation into the brains of immunocompromised rodents (Figure 1). Herein, the definition of the cancer stem cell will be reviewed and an alternative nomenclature proposed to enable future experimental study and clinical application.

### A matter of definition

The ability to develop consensus criteria for a “cancer stem cell” depends on which of the various stem cell populations we are describing. For this reason, it is important to distinguish between the particular stem cell subtypes relevant to glioma formation, maintenance, and progression.

### Glioma initiating stem cells (GISCs)

Emerging evidence from numerous laboratories using a diverse collection of genetically-engineered mouse (GEM) models supports the notion that low-grade and high-grade gliomas likely arise from neural stem cells (NSCs). These NSCs typically reside in germinal zones [19], such as the lateral ventricle subventricular zone (lv-SVZ) [20-21], the third ventricle (TVZ) [22-24], and the fourth ventricle [25-26]. Even within these ventricular zones, there are discrete subregions and cell types most capable of giving rise to glioma following the introduction of glioma-causing genetic changes (mutations) [23, 27-29]. In addition, some recent reports have suggested that not all gliomas arise from NSCs, such that gliomas can been initiated when astrocytes [30-31], oligodendrocyte progenitors [32-36], and even neurons [37] are targeted in experimental mouse model systems. While these more differentiated cell types do generate gliomas under specific conditions, it is not clear whether gliomagenesis requires a reversion to a more progenitor-like (stem cell) state from which glioma formation ensues [38]. Studies, such as those involving lineage tracing methods [33, 39], may help to resolve these issues. Nonetheless, glioma-initiating cells should have the same functional properties as the stem cells that normally reside in these germinal zones.

### Glioma maintaining stem cells (GMSCs)

From fully-formed human glioma specimens, cells have been isolated with properties typically attributed to stem cells [40-42]. In the case of high-grade gliomas, these cancer stem cells are capable of generating histologically-similar tumors following implantation into naïve rodent recipients (Koch's postulate). However, despite considerable effort, such cells have not been isolated from low-grade gliomas, raising intriguing questions about the microenvironmental conditions required for low-grade glioma establishment. In this regard, low-grade gliomas are highly dependent on their non-neoplastic microenvironment (stroma), and require microglia and other stromal cell types to initiate gliomagenesis and maintain tumor growth in GEM strains [43-45]: The failure to serially passage low-grade gliomas (e.g., pilocytic astrocytomas) using neurosphere preparations may not indicate an absence of GMSCs, but rather that the obligate stromal conditions are not accurately recapitulated in immunocompromised rats and mice. Alternatively, these low-grade glioma stem cells may harbor specific mutations that favor senescence, thus limiting their long-term maintenance [46].

Moreover, stem cells that derive from fully-formed cancers do not need to have the same biological properties as their normal NSC counterparts. In this respect, some laboratories have reported that GMSCs lack the capacity to give rise to all three CNS cell types (multi-lineage differentiation) as well as exhibit new properties not shared with normal NSCs (e.g., relative resistance to chemotherapy or radiation; [47-50]). For example, while normal NSCs are normally quiescent, cancer-maintaining stem cells in some situations can still proliferate [51-52]. In addition, there exists significant cancer stem cell heterogeneity in high-grade gliomas with respect to their cancer propagating ability [53]. Collectively, GMSCs represent those stem-like cells most capable of maintaining the tumor, such that their suppression limits glioma growth and increases the effectiveness of therapy.

### Glioma-associated stem cells (GASCs)

In addition to stem cells harboring initiating glioma-associated genetic mutations, there are also recruited stem cells in both human and experimental murine glioma tumors. These stem cells may originate from outside of the brain parenchyma (e.g., hematopoietic stem cells; [54]) or from stem cell niches within the CNS [55-56]. The homing of these stem cells to the developing tumor represents a natural response to CNS injury [57-58], such as occurs in the setting of cerebral ischemia (stroke; [59-61]) or multiple sclerosis (experimental allergic encephalomyelitis; [62-63]). The properties of these recruited stem cells thus reflect their region of origin (bone marrow, brain) in combination with remodeling that occurs as a result of adaptation to their newly adopted cellular environment. Aside from their potential therapeutic value as cellular payload delivery vehicles for oncolytic virus or chemotherapy [64-67], their function in glioma maintenance or response to treatment is unclear, with reports describing both glioma suppressing [68] and promoting [69] effects. Moreover, the markers used to define normal NSCs may not apply to those whose primary origins are outside of the brain.

### Knowing one when you see one

Current methods for identifying glioma stem cells rely mainly on protein marker expression and functional assessments. Each of these methods is valid, but both have limitations, which are important to consider when classifying the diverse cellular populations that contribute to gliomagenesis and progression.

### Cellular phenotyping

One of the criteria used to define glioma stem cells is the expression of stem cell markers, including CD133 (prominin-1), sox2, Olig2, nestin, brain lipid binding protein (BLBP), and CD44, which derive from studies on both CNS and non-CNS progenitor cell populations [70-71]. However, it should be appreciated that none of these markers exclusively identify stem cells and all are expressed in different CNS cell types at varying times during brain development. For example, Olig2 is also expressed in oligodendrocyte progenitor cells, nestin in reactive astrocytes, CD44 in astrocytes and microglia, BLBP in radial glia, and sox2 in oligodendrocytes. CD133 (prominin-1) has been uniformly used to mark glioma stem cells; yet, CD133-negative tumor cells have also been reported to generate tumors under specific conditions [72]. As mentioned above, it seems logical to develop antibody-based reagents that can be used to study each of the stem cell populations relevant to glioma pathogenesis [73-74]. To this end, we and others have recently employed GEM glioma models to identify transcripts unique to GMSCs relative to their non-neoplastic NSC counterparts (GISCs).

In addition, converging data from several laboratories have revealed striking heterogeneity in progenitor (stem) cells from different regions of the CNS [25, 75-76], raising the possibility that stem cell markers may differ depending on the brain region (cortex versus cerebellum) or developmental age (adult versus infant). For example, discrete gene expression patterns separate NSCs from the third and lateral ventricles [23] as well as neuroglial progenitors (radial glia) from the spinal cord and brain [78-79].

### Functional phenotyping

The lumping of all glioma-related stem cells into one group assumes that they are all functionally similar. Since the roles they play in glioma pathogenesis may be remarkably different (tumor initiation versus tumor maintenance), it is also likely that their biological properties will be dissimilar and reflect these distinct roles. For example, glioma-initiating stem cells should, by definition, have the capabilities of normal NSCs, since the genetic alterations that created the cancers occurred in these very cells. However, once the tumor is formed, those stem cells may no longer have the properties found in their normal counterparts, as they have sustained a series of mutations that could potentially reprogram them and limit the capabilities of the resulting stem cell progeny. In addition, for those cancers that arise following the introduction of genetic changes in more differentiated cells, it is possible that the reprogramming that ensued following the acquisition of the causative genetic changes results in cancer stem cells with properties more similar to de-differentiated cell types. Finally, as mentioned above, several studies using different experimental mouse glioma systems have revealed that NSCs from different locations within the CNS have differential capacities to increase their expansion and to form tumors following the introduction of glioma-associated genetic mutations [23, 77-79]. These findings further underscore the need to more fully appreciate the diversity of NSCs when characterizing glioma stem cell populations.

### Why it matters

**Figure 2:**
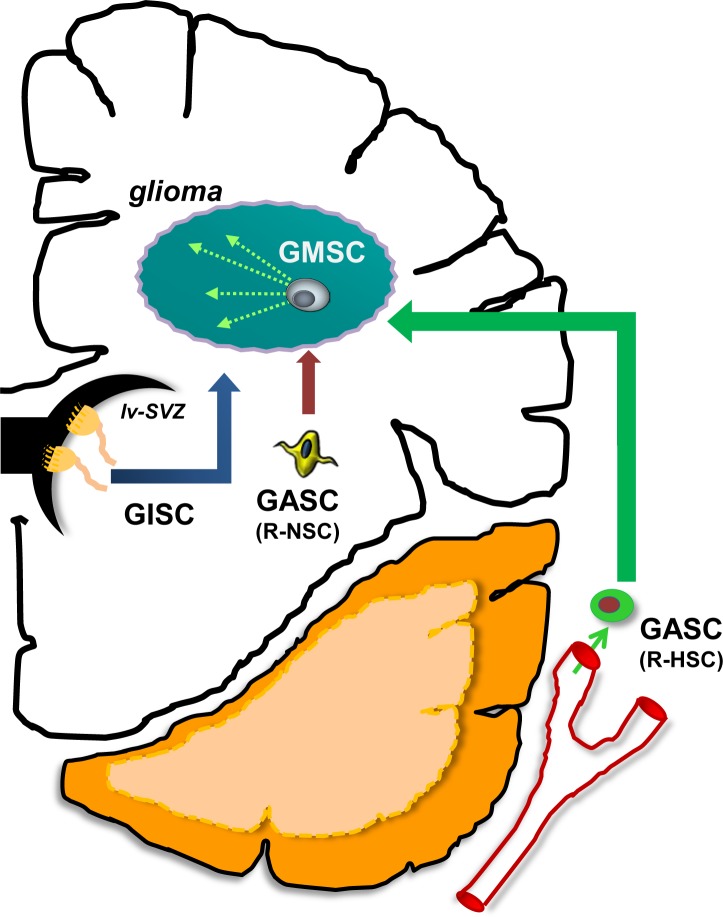
Several distinct populations of stem cells participate in glioma pathogenesis Glioma-initiating stem cells (GISCs) originate from germinal zones and serve as the cellular substrates for gliomagenesis following the acquisition of cancer-initiating genetic mutations. Glioma-maintaining stem cells (GMSCs) are isolated from mature gliomas and can propagate these tumors following transplantation into naïve recipient brains. Glioma-associated stem cells (GASCs) are recruited from local (brain) or distant (hematopoietic stem cells; HSCs) sites to populate the brain tumor.

While it may appear to be an epistemological debate, defining the particular glioma stem cell has profound implications for the types of questions to be addressed. Employing definitions relevant for GMSCs to the study of GISCs is likely to create interpretation issues and may result in inaccurate experimental conclusions. For this reason, we propose a taxonomy system to facilitate more meaningful scientific inquiry and lead to improved translational impact (Figure 2).

As such, studies focused on cell of origin (GISCs) should consider applying criteria used to characterize progenitor cells from that region of the CNS during the relevant developmental period. In this regard, tumor-initiating cell investigations require a more in-depth understanding of the particular germinal zone from which the specific glioma derives. For pilocytic astrocytomas, characterizing the stem cell niches relevant to pediatric gliomagenesis (third and fourth ventricular zones) as well as defining the innate and stroma-influenced capabilities of these progenitors during early life is critical. Similarly, in the case of adult high-grade gliomas, the focus would be on adult progenitor populations in the ventricular zones (*e.g*., lateral ventricle or other gliomagenic regions, such as the subcortical white matter) thought to give rise to these tumors. Analogous approaches would also be leveraged to define GASCs, based on their tissue of origin and the impact that the tumor has on the function of these recruited stem cells.

In contrast, stem cells from fully-formed tumors would be expected to have different properties than those that served as the cells of origin for that malignancy. During the process of gliomagenesis, those GMSCs will have acquired new phenotypes conferred both by the glioma-associated genetic mutations, but also by the unique microenvironment in which the glioma resides. To identify the proteins and biological functions unique to GMSCs will require comparisons not only to non-neoplastic primary NSCs containing these mutations, but also a more complete appreciation of how the glioma stem cell niche influences the epigenetic and gene expression profiles of these cells. A careful dissection of each of these stem cell populations offers new opportunities to more fully understand glioma formation, maintenance, and treatment.
